# Wnt5A-Mediated Actin Organization Regulates Host Response to Bacterial Pathogens and Non-Pathogens

**DOI:** 10.3389/fimmu.2020.628191

**Published:** 2021-02-16

**Authors:** Suborno Jati, Soham Sengupta, Malini Sen

**Affiliations:** Division of Cancer Biology and Inflammatory Disorder, CSIR-Indian Institute of Chemical Biology, Kolkata, India

**Keywords:** Wnt5A, actin, phagosome, pathogen, non-pathogen

## Abstract

Wnt5A signaling facilitates the killing of several bacterial pathogens, but not the non-pathogen *E. coli* DH5α. The basis of such pathogen vs. non-pathogen distinction is unclear. Accordingly, we analyzed the influence of Wnt5A signaling on pathogenic *E. coli* K1 in relation to non-pathogenic *E. coli* K12-MG1655 and *E. coli* DH5α eliminating interspecies variability from our study. Whereas cell internalized *E. coli* K1 disrupted cytoskeletal actin organization and multiplied during Wnt5A depletion, rWnt5A mediated activation revived cytoskeletal actin assembly facilitating K1 eradication. Cell internalized *E. coli* K12-MG1655 and *E. coli* DH5α, which did not perturb actin assembly appreciably, remained unaffected by rWnt5A treatment. Phagosomes prepared separately from Wnt5A conditioned medium treated K1 and K12-MG1655 infected macrophages revealed differences in the relative levels of actin and actin network promoting proteins, upholding that the Wnt5A-Actin axis operates differently for internalized pathogen and non-pathogen. Interestingly, exposure of rWnt5A treated K1 and K12-MG1655/DH5α infected macrophages to actin assembly inhibitors reversed the scenario, blocking killing of K1, yet promoting killing of both K12-MG1655 and DH5α. Taken together, our study illustrates that the state of activation of the Wnt5A/Actin axis in the context of the incumbent bacteria is crucial for directing host response to infection.

## Introduction

Wnt5A belongs to a 19-member family of Wnt ligands, which are secreted glycoproteins originally discovered with reference to embryonic development. Wnts interact with Frizzled (Fz) and ROR cell surface receptors. Frizzleds are seven transmembrane spanning receptors, about 12 in number, bearing homology to heterotrimeric G protein coupled receptors and RORs (ROR1 and ROR2) bear homology to tyrosine kinases (1–7). Classically, Wnt signaling is divided into two main categories – canonical (β-catenin dependent) and non-canonical (β-catenin independent) (6, 8). While canonical Wnt signaling mostly acts through β-catenin mediated transactivation of specific genes, non-canonical Wnt signaling often acts independent of β-catenin during regulation of cell polarity and differentiation (9–12). On account of sequence homology within the Wnt and Frizzled/ROR family members, cross reactivity in Wnt-Frizzled/ROR interactions and crosstalk among the signaling intermediates of the canonical and non-canonical Wnt signaling pathways is quite frequent (7, 13, 14).

Wnt5A, a prototype of the non-canonical Wnt signaling pathway interacts with Fz2, Fz4, Fz5 and ROR1/2 receptors regulating cell polarity and movements (2, 4, 5). Quite naturally, Wnt5A signaling is an important facet of macrophages, which respond to a broad spectrum of environmental cues including bacterial infections, through alterations in cell migration and polarity (15–17).

Macrophages are intrinsically wired to counter bacterial infections through phagocytosis, which utilizes the coordination of the actin cytoskeleton with different signals (17–19). While some pathogenic bacteria fall prey to macrophages, others escape the immune defense program therein through either self-extrusion or creation of a protective intracellular niche (20, 21). Several other bacteria, mostly non-pathogenic commensals are also able to reside in macrophages without being killed (22, 23). Thus bacterial infections are inherently associated with the cytoskeletal actin dynamics of macrophages (24–26). However, despite considerable research and extensive knowledge in this field our understanding of how host defense mechanisms influence the cytoskeletal actin organization to regulate infection outcome remains incomplete.

Several labs including ours’ have demonstrated that Wnt5A signaling induces alterations in actin assembly in macrophages (16, 24, 27, 28). This finding is in perfect agreement with the depicted role of Wnt5A in bacterial phagocytosis (15). Wnt5A induced alterations in actin assembly are in fact linked with a Rac1-Disheveled dependent host autophagy circuit that promotes both internalization and killing of pathogenic bacteria such as *Pseudomonas* sp., which are associated with respiratory disorders (16). Wnt5A mediated killing of *Mycobacterium* sp. through the host autophagy machinery has also been demonstrated (29). Interestingly, however, non-pathogenic *E. coli* are internalized by Wnt5A signaling, but not killed (15), this being in line with the reported survival of non-pathogenic *E. coli* in macrophages through extended time periods (30, 31). These differences in infection outcome led us to investigate how Wnt5A aided actin assembly controls different bacterial infections at the molecular level.

In the current report we demonstrated using pathogenic *E. coli* K1 and non-pathogenic *E. coli* K12-MG1655 and *E. coli* DH5α that the outcome of Wnt5A assisted actin organization is different for the pathogenic and non-pathogenic strains of *E. coli*. While activation of actin assembly by Wnt5A facilitated the killing of only the pathogenic but not the non-pathogenic *E. coli*, intercepting activation of the Wnt5A-Actin axis by actin assembly inhibitors reversed the scenario, leading to elimination of the non-pathogen but not the pathogen. Overall, our data indicate that Wnt5A signaling controls the outcome of different bacterial infections at least partly through actin organization.

## Materials and Methods

### Cell Culture and Reagents

RAW 264.7 macrophages (ATCC^®^ TIB71™), and mouse peritoneal macrophages were maintained under normal tissue culture conditions following published protocol (32). *E. coli* K1 (gift from Dr. Victor Nizet, UCSD, CA), *Pseudomonas aeruginosa* strain PA14 (gift from Dr. Chitra Mandal, IICB, Kolkata), *E. coli* DH5α and *E. coli* K12-MG1655 (MTCC. 1586) were used to infect RAW 264.7 and peritoneal macrophages. PIPES (P1851), EGTA (E3889), Glycerol (G5516), ATP (A2383), NaCl (S5886), NP-40 (492018), Triton X-100 (11332481001), and Anti-Mouse HRP (A4416) were purchased from Sigma-Aldrich (St. Louis, MO, USA). RNAiMax Transfection Reagent (13778150) was purchased from Invitrogen (Thermo Fisher Scientific, Waltham, MA, USA). Phalloidin (A34055) and DAPI (D1306) were purchased from Molecular Probes (Eugene, OR, USA). rWnt5A (GF146), Rac1 inhibitor (NSC23766), Arp-2/3 complex inhibitor I (CK-666) & II (CK-869), Tris base (648310), and Na_3_VO_4_ (D00152519) were purchased from Calbiochem (San Diego, CA, USA). MgCl_2_ (60583305001046), Tween 20 (655205), β-mercaptoethanol (8057400250), NaF (61773705001730), PVDF membrane (IPVH00010) and Luminataclassico chemiluminescent substrate (WBLUC0500) were purchased from Millipore (Burlington, MA, USA). Anti-Wnt5A (MAB645) and Anti- Rat HRP (HAF005) antibodies were purchased from R&D (Minneapolis, MN, USA). Anti-Actin antibody (ACTN05-C4) was purchased from Thermo Fisher Scientific (Waltham, MA, USA). On-target plus SMART Pool siRNA against murine Wnt5A (L-065884-01) and nontargeting pool control siRNA (D-001810-05) were purchased from Dharmacon (Lafayette, CO, USA). Anti-β-actin (SC-47778), Rac1 (SC-217), and Rab7 (SC-376362) antibodies were purchased from Santa Cruz Biotechnologies (Dallas, TX, USA). Anti-Arp2 (Thr-237/Thr-238), phospho-specific (AP3871), and Anti-Arp2 (AP3861) antibodies were purchased from ECM Biosciences (Versailles, KY, USA). DMSO (196055) was purchased from MP-Biomedicals (Solon, OH, USA). cDNA synthesis kit and Taq Polymerase were purchased from BioBharati Life Sciences (Kolkata, India). RNA IsoPlus was purchased from Takara (Kusatsu, Shiga, Japan). Mouse Wnt5a: 5′-CAGGTCAACAGCCGCTTCAAC-3′ (forward) 5′-ACAATCTCCGTGCACTTCTTGC-3′ (reverse) and GAPDH: 5′-ACCACAGTCCATGCCATCAC-3′ (forward); 5′-TCCACCACCCTGTTGCTGTA-3′ (reverse) primers was purchased from Integrated DNA Technologies, USA were used to conduct RT-PCR.

### Bacterial Killing Assay

Cells grown to about 60% confluency were infected separately with different bacteria at MOI: 10 for 1 h (T0), after which the extracellular bacteria were discarded by extensive washing. Infected cells were incubated from 1 to 4 h (T1-T4) under normal tissue culture conditions, harvested, lysed in autoclaved distilled water and plated on agar plates for CFU (Colony Forming Units) enumeration.

For the inhibitor assay cells were infected with different bacteria for 1 h and post-infection different inhibitors were added to the media after PBS washes and kept for 3 h. CFU enumeration was done both at 1 h (initial) and 3 h (final) time points. Percentage of bacteria killed was calculated by the equation: [(Initial CFU − Final CFU)/Initial CFU] × 100. Calculations were controlled to cell number.

### Western Blotting

Harvested cell pellet was lysed using cell lysis buffer for 15 min at 4°C (16). Following centrifugation at 12,000 rpm for 5 min at 4°C, about 40 μg of the clear lysate was run on SDS-PAGE and transferred to PVDF membrane. After blocking with 5% BSA for 2 h the membrane was incubated with primary antibody at 4°C, following which appropriate HRP-secondary antibody was added and incubation continued at room temperature. Finally, the membrane was visualized by Chemi documentation system of Azure Biosystems, Model‐C400. GelQuant.Net was used for calculation of band intensities.

### Transfection

Wnt5A siRNA transfection was done as reported previously (32). Briefly, RAW264.7 macrophages were plated in six-well tissue culture plates (~2 × 10^6^ cells per well) a day before transfection and incubated at 37°C in 5% CO_2_. On day of transfection, initially 0.7 ml of medium containing 2% FBS was added to the cells. Subsequently 25 nM Wnt5A siRNA or random siRNA was complexed with 5 μl of Lipofectamine RNAiMax transfection reagent in 300 μl of antibiotic-free serum-free culture medium and incubated for 30 min before adding to the cells. The cells were incubated for 24 h after which the culture medium was replaced with complete medium containing antibiotic and incubated for about 32 h. Transfected cells were infected with bacteria for 1 h (T0), following which, the infection was removed and cells were kept for 3 h (T3) under normal tissue culture condition without antibiotic. CFU was plotted controlled to cell number.

### Filamentous (F) Actin Preparation and Immunoblotting

F-actin isolation was done following published protocol (33). Briefly, cells were harvested, resuspended in F-actin Stabilization Buffer (FSB) (33) and kept at 37°C for 10 min following which the mix was centrifuged at 2,000 rpm to separate the debris and unbroken cells. The supernatant was centrifuged at 150,000*g* for 60 min in SW61 rotor to obtain the F-actin pellet, which was resuspended in F-actin destabilizing solution (10 µM Cytochalasin D in sterile distilled water). F-actin level was estimated by immunoblotting with actin antibody.

### Phagosome Isolation

Phagosome was isolated following published protocol (16). RAW264.7 cells pretreated with Wnt5A conditioned medium (L5A) and control medium (L) from L cells (32) for 6 h were infected with bacteria (MOI: 10) for 1 h without added antibiotic. After removal of bacteria and addition of fresh DMEM, incubation of infected cells was continued for 2 additional h following which the harvested cells were washed with ice cold PBS (twice), resuspended in homogenization buffer [HB; 20 mM HEPES/KOH (pH 7.2), 0.5 mM EGTA, and 250 mM sucrose] and left on ice for 5 min. Following centrifugation at 2,000 rpm the cell pellet was resuspended in 2 ml HB (without EGTA) and lysed in a dounce homogenizer. After another centrifugation at 440*g* for 3 min at 4°C, 2.4 ml of 65% sucrose was added to the 2 ml clear homogenate to obtain 39% final sucrose concentration. This 4.4 ml sucrose containing cell homogenate was layered over a discontinuous sucrose gradient made with 1 ml 65% sucrose and 2 ml 55% sucrose. Subsequently, 2 ml of 32.5% sucrose and 1 ml of 10% sucrose were added on top of the cell homogenate layer. This was followed by ultracentrifugation in SW41 rotor at 100000 X *g* for 1 h. Phagosome was collected from the interface of 55% and 65% sucrose layers, pelleted by centrifugation at 18,000 X *g* for 10 min and further resuspended in HB. The resuspended phagosome pellet was boiled at 95°C for 10 min in SDS-PAGE 4X sample buffer for immunoblotting. During inhibitor experiment, the inhibitor was added to cells after 1 h of infection with the bacteria. Following 3 h incubation after removal of the bacteria, phagosome was prepared. For phagosome CFU calculation, phagosome pellet was resuspended in HB and 3 µl of the suspension was added to 50 µl of autoclaved distilled water. 1:20 dilution in fresh autoclaved distilled water was plated on LB Agar plate. For western blotting, sample preparation was done by addition of 4X sample buffer to equal volumes of resuspended phagosome, followed by boiling at 95°C for 15 min.

### Confocal Microscopy

Confocal microscopy was done following previously published protocol (16). Briefly peritoneal macrophages and RAW264.7 cells were plated onto three chambered glass slides. Fixed cells (fixation was done in 4% paraformaldehyde for 15min) were stained with Phalloidin (Alexa Fluor 455, 1:2,000) and DAPI (1:4,000) in 2.5% BSA dissolved in PBST (0.1% Tween-20) for 15 min, which was followed by 3× PBST washes. The slides were mounted and visualized under Olympus Fluoview FV10i at 60x objective and 1.6× zoom. Fluorescence intensity was measured by ImageJ.

### Statistical Analysis

Results were analyzed with unpaired Student *t* test using Graph- Pad Prism 6 software. Line diagrams and bar graphs are expressed as mean ± SEM. *p* ≤ 0.05 is considered statistically significant. Significance is annotated as follows: *p ≤ 0.05, **p ≤ 0.005, ***p ≤ 0.0005.

### Ethics Statement

All animal studies were approved by the Animal Ethics Committee of CSIR-IICB.

## Results

### Wnt5A Signaling Interconnects Differently With Pathogenic and Non-Pathogenic *E. coli*

**Figure 1:**
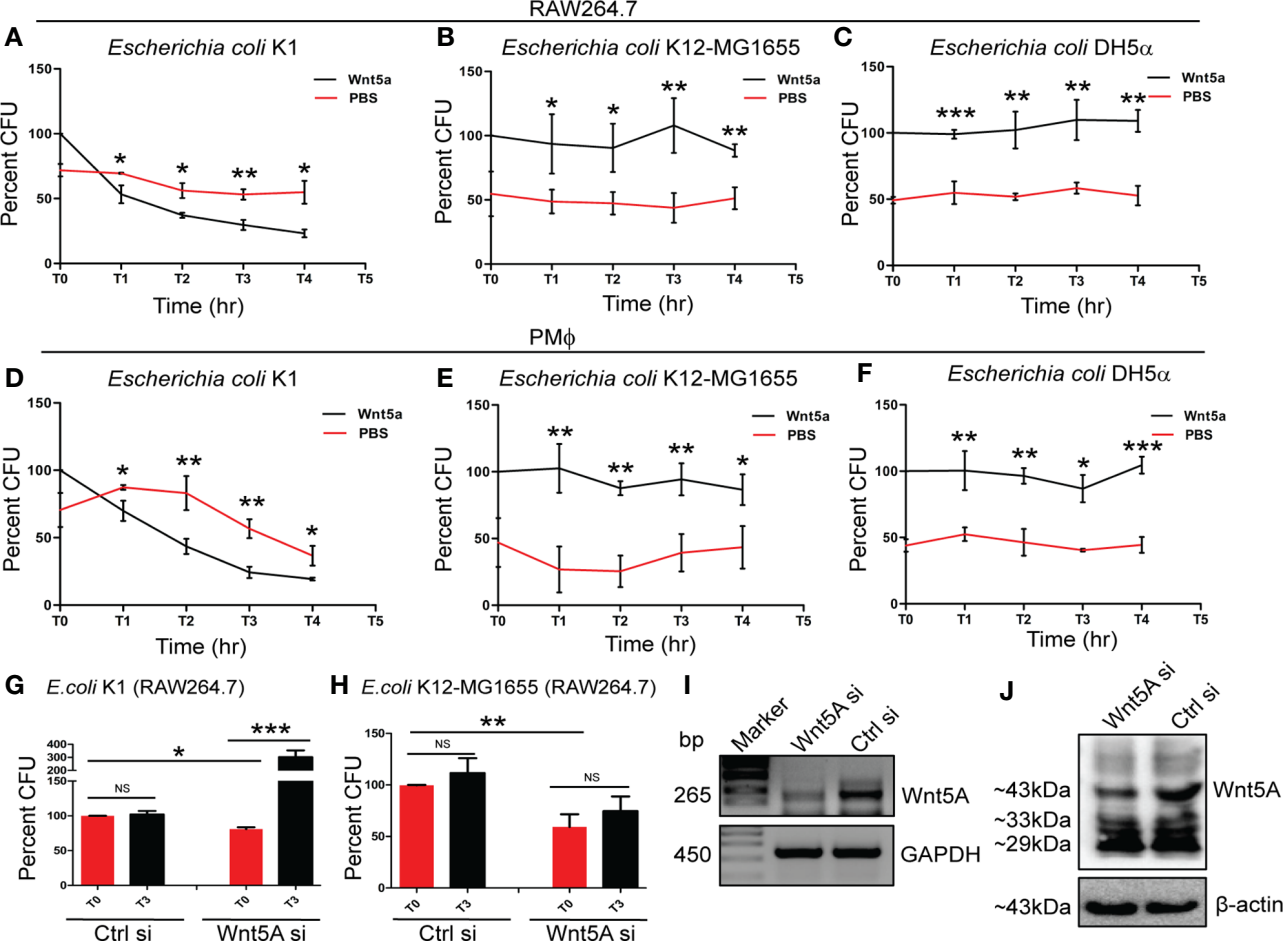
Wnt5A signaling facilitates killing of pathogenic but not non-pathogenic *E. coli*. rWnt5A promoted intracellular killing of pathogenic bacterial strain *E. coli* K1 in both RAW264.7 **(A)** and peritoneal macrophages: PMϕ **(D)** as estimated by Colony Forming Units (CFU) (n = 3) at different time points (T1-T4), 1 h after infection (T0) at MOI: 10, as compared to corresponding control (PBS). rWnt5A did not promote killing of non-pathogenic bacterial strains *E. coli* K12-MG1655 **(B, E)** (n = 3) and *E. coli* DH5α **(C, F)** (n = 3) as observed in both RAW264.7 and PMϕ in comparison to corresponding control. The CFU obtained at T0 for Wnt5A treated set is considered 100 and the values in other points are normalized accordingly. Wnt5A siRNA mediated decrease in endogenous Wnt5A expression resulted in decreased uptake of both *E. coli* K1 and *E. coli* K12-MG1655, yet promoted intracellular proliferation of pathogenic *E. coli* K1 (n = 3) but not non-pathogenic *E. coli* K12-MG1655 (n = 3) as depicted by CFU at T0 and T3 **(G, H)**. Depletion of Wnt5A expression by siRNA was confirmed by RT-PCR **(I)** and immunoblot analysis **(J)** in RAW264.7 cells. Data represented as mean ± SEM; *p ≤ 0.05, **p ≤ 0.005, ***p ≤ 0.0005; NS, not significant. Wnt5Asi, Wnt5A siRNA; Crtlsi, control siRNA; Marker, DNA ladder.

In order to compare the effect of Wnt5A signaling on bacterial pathogens and non-pathogens, we focused on *E. coli* K1 as a pathogen in relation to *E. coli* K12-MG1655 or *E. coli* DH5α as non-pathogen. Genome comparisons reveal 19 islands, which code for different virulent factors in *E. coli* K1 but not *E. coli* K12-MG1655 or *E. coli* DH5α (34–39). Thus, to eliminate interspecies variability from our study, these bacteria were used as pathogen and non-pathogen. Wnt5A signaling was activated both in RAW 264.7 and mouse peritoneal macrophages by recombinant Wnt5A (rWnt5A) treatment (50 ng/ml) for 6 h prior to bacterial infection (15, 16, 27). Subsequently, intracellular bacterial killing vs. survival was estimated by comparing the CFUs retrieved after infection for 1 h (T0) with those retrieved at 1-h intervals (T1–T4) during incubation of the cells post infection. CFU (T0) of *E. coli* K1 was always higher than that of *E. coli* K12-MG1655 or *E. coli* DH5α perhaps on account of the invasiveness of this pathogenic strain. Interestingly however, in both cell types activation of Wnt5A signaling by rWnt5A increased intracellular killing of *E. coli* K1 as compared to the corresponding control (PBS). The internalized non-pathogenic strains K12MG1655 and DH5α on the other hand, were not killed by rWnt5A (**Figures 1A–F**). In agreement with these findings, while *E. coli* K1 multiplied intracellularly upon siRNA mediated Wnt5A depletion, there was no significant multiplication of the non-pathogenic strain K12MG1655 under similar condition (**Figures 1G, H** **)**. About 50% depletion of Wnt5A mRNA and protein upon siRNA transfection is depicted in panels I, J.

### Interrelation Between Wnt5A Signaling and Pathogenic or Non-Pathogenic *E. coli* Is Associated With Actin Assembly

The intrinsic association of cytoskeletal actin with bacterial infections (19, 40) led us to investigate if killing vs. survival of the pathogenic and non-pathogenic *E. coli* by Wnt5A signaling is associated with actin assembly.

Accordingly, we first studied the effect of the bacterial infections on actin assembly and then examined if activation of Wnt5A signaling in the infected cells introduces alterations in the scenario. Filamentous (F) actin was estimated for analyzing the extent of actin assembly. Initially, both uninfected and infected mouse peritoneal macrophages were stained with phalloidin, which binds to F-actin and visualized by confocal microscopy. As an alternative measure, F-actin of uninfected and infected macrophages was separately isolated through ultracentrifugation of suspensions of the broken cells in F-actin stabilzation buffer (33) and quantified by immunoblotting.

**Figure 2:**
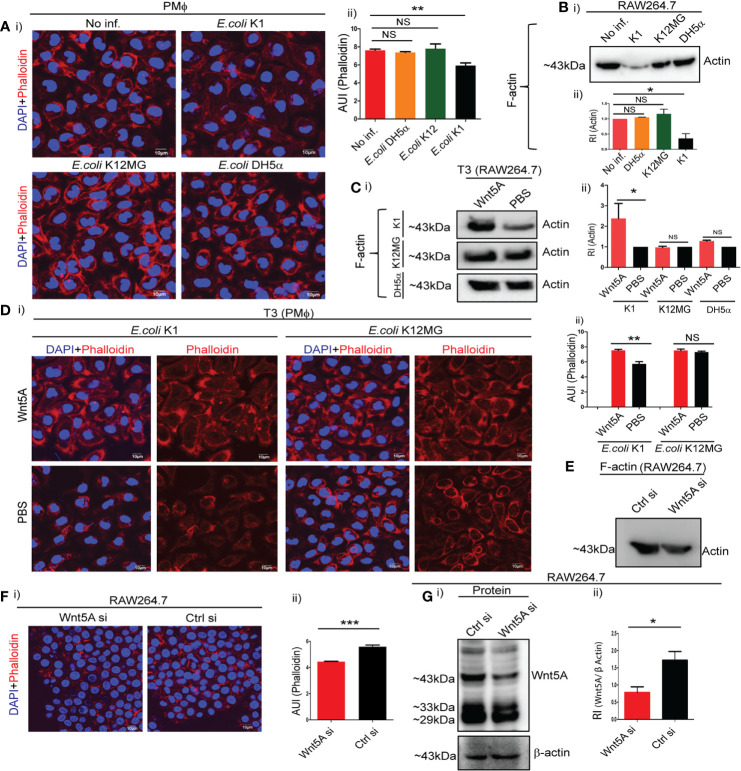
Wnt5A signaling alters cytoskeletal actin assembly in relation to bacterial infection. Infection of PMϕ and RAW264.7 by pathogenic *E. coli* K1, but not non-pathogenic *E. coli* K12-MG1655 or *E. coli* DH5α, at MOI:10 for 1 h resulted in decrease of total cellular F-actin as depicted by confocal microscopy of phalloidin stained cells **(A**: i, ii**)** and immunoblotting of isolated F-actin **(B**: i, ii**)** (n = 3). Phalloidin intensity (microscopy) and actin band intensity (immunoblotting) were measured by ImageJ and GelQuant (densitometry), respectively. Wnt5A signaling opposed effect of K1 infection (3 h incubation after 1 h infection: T3), enhancing F-actin formation as observed by immunoblotting **(C:** i, ii**)** and confocal microscopy of phalloidin stained cells **(D**: i, ii**)** (n = 3). Wnt5A signaling produced little or no change in F actin upon *E. coli* K12-MG1655 and *E. coli* DH5α infection following similar procedure **(C, D)**. Decrease in endogenous Wnt5A level resulted in decrease of total cellular F-actin in RAW 264.7 as demonstrated by immunoblotting **(E)** and confocal microscopy **(F**: i, ii**)** (n = 3). Depletion of Wnt5A expression by siRNA transfection was assessed through immunoblotting **(G**: i, ii**)**. Data represented as mean ± SEM; *p ≤ 0.05, **p ≤ 0.005, ***p ≤ 0.0005; NS, not significant. Phalloidin stain shown in red and DAPI (nuclear) stain shown in blue. AUI, arbitary unit of intensity; RI, relative intensity.

Confocal microscopy of phalloidin stained peritoneal macrophages after bacterial infection for 1 h revealed significant reduction in F-actin by *E. coli* K1, but not by K12-MG1655 and DH5α. Intensity of phalloidin stain as a measure of F-actin assembly was estimated by Image J analysis (**Figure 2A**, i and ii). These findings were corroborated by immunoblotting of F-actin using RAW 264.7 macrophages. Infection of RAW264.7 cells with K1 but not K12-MG1655 and DH5α led to significant reduction in the level of F-actin (**Figure 2B**, i and ii). Activation of Wnt5A signaling through added rWnt5A (using PBS as vehicle control), on the other hand, led to significant increase in assembled actin (F-actin) in K1 infected but not K12-MG1655 or DH5α infected RAW264.7 cells during 3 h post infection (T3) (**Figure 2C**, i and ii), as demonstrated by immunoblotting. Phalloidin staining followed by confocal microscopy of peritoneal macrophages infected with either K1 or K12-MG1655 after activation of Wnt5A signaling yielded the same results as projected by Image J analysis (**Figure 2D**, i and ii). Thus, while significant increase in actin assembly by Wnt5A as compared to control (PBS) in the case of *E. coli* K1 infection correlated with appreciable bacterial killing, no significant change in actin assembly by Wnt5A in the case of K12-MG1655 or DH5α infection correlated with survival.

That Wnt5A signaling inherently promotes actin assembly was validated by the reduced level of F-actin in Wnt5A depleted macrophages, as demonstrated by both immunoblotting and confocal microscopy (**Figures 2E, F** **)**. **Figure 2G** demonstrates the siRNA-mediated reduction in Wnt5A level as compared to control. Accordingly, activation of the Wnt5A-Actin axis countered the disruptive effect of *E. coli* K1 on assembled actin. Since K12-MG1655 and DH5α are not detrimental to assembled actin, there was no major influence of Wnt5A signaling on actin assembly in the cells infected with these strains.

### Different Phagosome Compositions of the Pathogen and Non-Pathogen Infected Macrophages Reflect Difference in Their Actin Organizations

Cytoskeletal actin dynamics during bacterial internalization result in phagosome formation (41, 42). Since phagosomes control the fate of internalized bacteria, we examined if the respective phagosome compositions associated with pathogenic (K1) and non-pathogenic (K12-MG1655) *E. coli* infections in Wnt5A activated macrophages feature the observed differences in actin assembly (**Figure 2**). Phagosomes were harvested separately from similar numbers of *E. coli* K1 and *E. coli* K12-MG1655 infected RAW 264.7 macrophages 3 h post infection and analyzed by immunoblotting (16). Prior to infection, the macrophages were activated with either Wnt5A conditioned medium prepared from Wnt5A overexpressing L cells (L5A) or treated with L cell conditioned medium (L) as control. Wnt5A conditioned medium, as a source of Wnt5A (15, 16, 32), was used to limit the use of the expensive rWnt5A protein.

**Figure 3:**
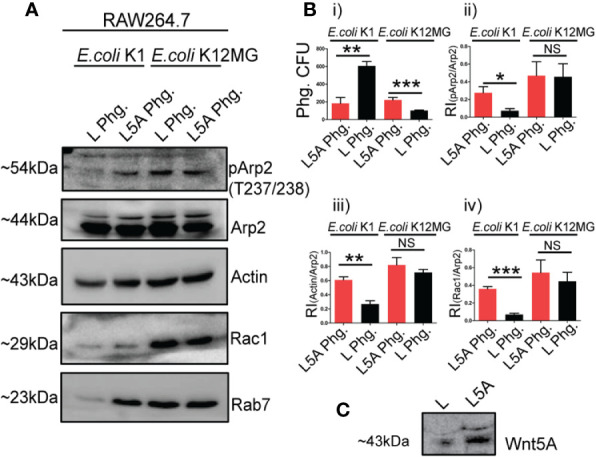
Wnt5A (L5A) signaling aided actin assembly during pathogen vs. non-pathogen infection relates to altered phagosomal composition. Levels of phosphorylated Arp2 (pArp2), Actin, Rac1 and Rab7 with reference to unphosphorylated Arp2 was higher in phagosome of L5A treated *E. coli* K1 infected RAW264.7 macrophages (L5A Phg) 3 h post infection in comparison with the control phagosome from L treated infected RAW264.7 (L Phg.) as demonstrated by immunoblotting (**A**: lanes 1, 2) (n = 3). In *E. coli* K12-MG1655 infection there was no such difference in the level of pArp2, Actin, Rac1, Rab7 between L5A Phg. and L Phg. (**A**: lanes 3, 4). CFU enumeration demonstrated L5A mediated killing of K1 but not K12-MG1655 at phagosome level **(B**.i**)** (n = 3). Bii, Biii and Biv depict the Relative Intensity (RI) of pArp2, Actin and Rac1 in relation to unphosphorylated Arp2 in L5A Phg. and L Phg of the *E. coli* K1 and *E. coli* K12-MG1655 infected sets. **(C)** depicts the presence of Wnt5A in L5A conditioned media (L-cells stably expressing Wnt5A) but not in L conditioned media by immunoblotting. Data represented as mean ± SEM; *p ≤ 0.05, **p ≤ 0.005, ***p ≤ 0.0005; NS, not significant.

Indeed, in case of *E. coli* K1 infection, phagosomes of L5A treated cells assembled significantly more actin than those of the L treated cells, substantiating increased Wnt5A assisted actin assembly in K1 infection. L5A induced increase in phagosomal actin correlated with augmented accumulation of phosphorylated Arp2 (p-Arp2: phosphorylated at Thr 237/238, Tyr.202), which is crucial for initiation of actin polymerization (43), but not unphosphorylated Arp2. Another regulator of actin assembly, Rac1 (44, 45), also accumulated more in the L5A phagosomes than the L phagosomes of the K1 set. L5A mediated increased phagosomal actin assembly also correlated with phagosomal maturation as depicted by increase in Rab7, a marker of phagolysosomes (**Figure 3A**, lanes 1, 2). This result was in accordance with increased K1 killing, i.e. lesser number of K1 in the L5A phagosomes as compared to the L phagosomes (**Figure 3B** **, i**). In case of *E. coli* K12-MG1655 infection, no difference in actin, Rac1, p-Arp2 or Arp2 between the L5A and L phagosomes was noted, supporting lack of significant alteration of actin assembly (**Figure 3A**, lanes 3, 4). In addition, there was also no increase in phagosomal maturation and activity upon stimulation with L5A, as evident from the similar number of retrieved K12-MG1655 from the L5A and L phagosomes (**Figures 3A, B**, i). Panels Bii, Biii & Biv depict the L5A induced increase in phagosomal p-Arp2, actin and Rac1 with reference to unphosphorylated Arp2 in the K1 but not K12-MG1655 infected macrophages, validating the occurrence of altered actin dynamics during Wnt5A assisted bacterial killing. Panel C denotes presence of Wnt5A in L5A conditioned medium but not in L.

### Inhibitors of Actin Assembly Alter the Fate of *E. coli* K1 and *E. coli* K12MG1655/*E. coli* DH5α Infections

To separately validate that Wnt5A aided actin assembly is intrinsically associated with the outcome of infections with pathogenic and non-pathogenic *E. coli* we examined if the effect of these bacterial infections can be altered through the application of actin assembly inhibitors, which block activation of Arp2/3 complex and Rac1, thereby inhibiting actin nucleation and branching (46, 47).

Wnt5A or PBS (vehicle control) pretreated RAW 264.7 and mouse peritoneal macrophages were infected with either *E. coli* K1 (pathogenic) or K12-MG1655 and DH5α (non-pathogenic) for 1 h, washed free of extracellular bacteria and incubated for 3 h with inhibitors to Arp2/3 complex (20 µM) and Rac1 (15 µM) using DMSO and PBS as vehicle controls, respectively (46, 47). Bacterial CFU retrieved from the infected cells before and after 3 h incubation depicted the effect of inhibition of Wnt5A assisted actin assembly on infection outcome. Confocal microscopy of the phallodin stained cells was performed to validate inhibitor - induced alteration in actin assembly.

**Figure 4:**
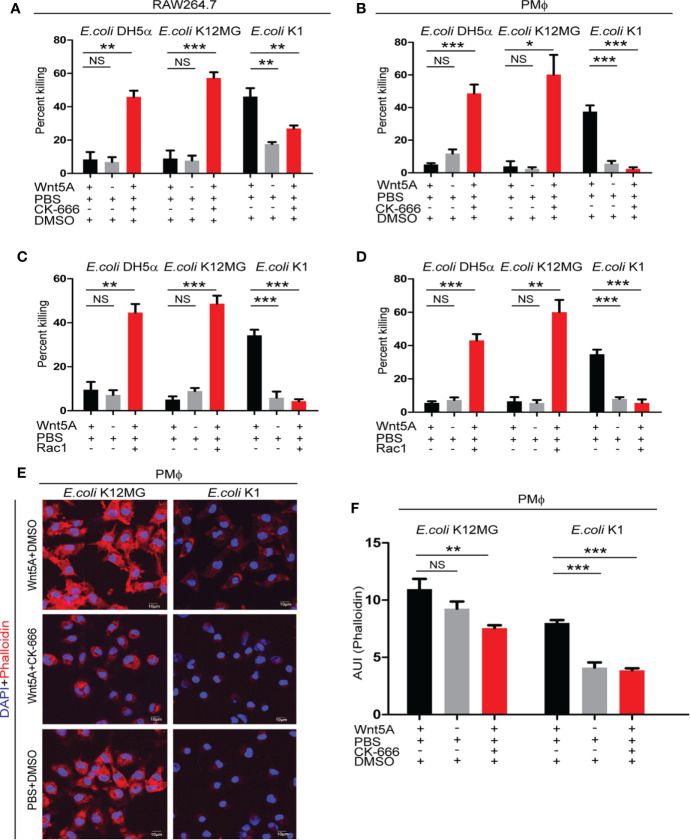
Arp2/3 complex and Rac1 inhibitors modify the Wnt5A induced fate of pathogenic and non-pathogenic bacteria by inducing cytoskeletal actin assembly in infected macrophages. Class I Arp2/3 complex inhibitor (CK-666; 20 µM) (n = 3) and Rac1 inhibitor (Rac1; 15 µM) (n = 3) treatment post infection promoted killing of *E. coli* K12MG1655 (*E. coli* K12MG) and *E. coli* DH5α in Wnt5A activated cells but impaired the killing of *E. coli* K1 in both RAW 264.7and peritoneal macrophages as presented in **(A–D)**. Arp2/3 complex Class I inhibitor (CK-666; 20 µM) altered Wnt5A induced actin modulation both in case of *E. coli* K12MG1655 and *E. coli* K1 as detected by phalloidin staining in peritoneal macrophages and confocal microscopy. F actin organization of Wnt5A+CK-666 added *E. coli* K12MG set was more than that of the Wnt5A+CK-666 added *E. coli* K1 set **(E, F)** (n = 4). DMSO+PBS was used as vehicle control for the experiment. Data represented as mean ± SEM; *p ≤ 0.05, **p ≤ 0.005, ***p ≤ 0.0005; NS, not significant.

Interestingly, Arp2/3 complex (CK666 & CK869) and Rac1 inhibitors blocked killing of *E. coli* K1 but promoted killing of both K12-MG1655 and DH5α in Wnt5A activated RAW 264.7 and mouse peritoneal macrophages (**Figures 4A–D** and **Supplementary Figures 1A, B**). No notable effect of the inhibitors on the infection load in PBS (control for Wnt5A) treated macrophages suggested that the inhibitors were active only when actin assembly was stimulated by Wnt5A signaling (**Supplementary Figures 2A, B**). From confocal microscopy of phalloidin stained cells it was evident that the inhibitors significantly reduced actin assembly (phalloidin stain) in the Wnt5A activated K1 and K12-MG1655 infected macrophages. However, while in case of K1, the inhibition resulted in a rather low level of actin assembly, similar to the control (PBS set), in case of K12-MG1655 considerable actin assembly persisted even after the inhibition (**Figures 4E, F** **)**. As before, there was no significant effect on phalloidin stain in absence of stimulation by Wnt5A (**Supplementary Figures 2C, D**).

Differences in actin assembly in the inhibitor treated MG1655 and K1 infected macrophages were indeed reflected also at the phagosome level, Rab7 indicating phagosome maturation. However, although the inhibitors diminished actin assembly, there was relatively more actin, p-Arp2 and Rac1 in relation to unphosphorylated Arp2 in the MG1655 phagosomes as compared to the K1 phagosomes (**Supplementary Figure 3**).

Unlike K1 infection, which antagonizes actin assembly, K12-MG1655 infection does not have appreciable influence on actin assembly (**Figure 2**). Accordingly, the residual assembled actin after administration of actin assembly inhibitors was always notably more in K12-MG1655 infected macrophages as compared to K1 infected macrophages.

These results clearly indicate that optimal conditions with regard to actin assembly are required for the killing of pathogens and non-pathogens. In case of K1 infection, the optimal condition was obtained through activation of Wnt5A signaling, but in case of K12-MG1655 or DH5α infection, additional influence of actin assembly inhibitor was required.

## Discussion

Host-pathogen interactions focusing on how various bacterial pathogen specific virulence factors hijack the actin cytoskeleton have been extensively described (19, 25). But how the host defense system incorporates the actin network to counter bacterial infections remains unclear. How bacterial non-pathogens as compared to pathogens fit in this scenario is also not clearly understood.

In view of the role of Wnt5A signaling in actin assembly (16, 24, 27, 28), we studied how the Wnt5A–Actin axis regulates the outcome of infection by a bacterial pathogen (*E. coli* K1 isolate from a biliary sepsis patient) as compared to a non-pathogen (lab strain *E. coli* DH5α or *E. coli* K12-MG1655). The basis of pathogenicity and non-pathogenicity of these *E. coli* strains have already been documented (36–39). Thus we did not focus on how particular virulence factors affect the actin cytoskeleton during infection. Rather, we studied the influence of the Wnt5A-Actin axis on the pathogen and the non-pathogen, and vice versa.

We observed that the pathogen antagonizes actin assembly in macrophages and disrupts it, as depicted by biochemical estimation of cellular F-actin and confocal microscopy of phalloidin stained cells. But activation of Wnt5A mediated actin organization in the pathogen-infected macrophages, as ascertained by similar methodologies including analysis of phagosome composition, promotes killing of the pathogen (**Figures 1**, **2**, and **3**). Application of actin assembly inhibitors, moreover, blocks the killing promoted by Wnt5A signaling (**Figure 4** and **Supplementary Figure 1**). On the contrary, the non-pathogens, which have no significant effect on Wnt5A mediated actin organization, remain protected by Wnt5A (**Figures 1**, **2**, and **3**). Consequently, diminution in Wnt5A assisted actin organization by actin assembly inhibitors promotes killing of the non-pathogens (**Figure 4** and **Supplementary Figure 1**). Thus, as explained in a simple model (**Supplementary Figure 4**), it is the extent and type of actin organization in relation to the incumbent bacteria, which decides whether the bacteria will be killed, Wnt5A signaling being a significant player in this interplay. *E. coli* K12-MG1655 is protected by host Wnt5A signaling because this non-pathogenic strain is compatible with Wnt5A induced actin alteration. Conversely Wnt5A signaling opposes infection by *E. coli* K1 facilitating its clearance because K1 is incompatible with Wnt5A signaling and decreases F-actin assembly. Thus, it may be stated that bacterial infections in macrophages can be managed through changes in the degree of actin assembly by regulation of Wnt5A signaling through activation by rWnt5A and application of specific inhibitors at appropriate dosages. Accordingly, Wnt5A signaling may be envisaged as a regulator of immune resistance to harmful infections. This concept is corroborated by the regulatory effect of Wnt5A signaling on infections by pathogenic bacteria such as *Streptococcus pneumoniae*, *Pseudomonas aeruginosa*, and *Mycobacterium tuberculosis* (16, 29). Incidentally, like K1 infection, infection by *Pseudomonas aeruginosa* (PA14) also causes decrease in polymerized actin (**Supplementary Figure 5**), and activation of Wnt5A signaling leads to bacterial killing (16). In connection with this study it is to be noted that Wnt5A signaling promotes the survival of the pathogen *E. chaffensis* (48). Hence it is important to look into the interrelation between Wnt5A assisted actin assembly and *E. chaffensis* infection.

Many aspects of host-mediated regulation of bacterial infections may be associated with cytoskeletal actin. These, may very well involve several TLRs, NODs, cholesterol and other lipids, and several components of the host autophagy machinery (19, 49–51). For example, Pathogen Associated Molecular Patterns (PAMP) like LPS and CpG-rich bacterial DNA can facilitate actin assembly and polarity of macrophages by binding with TLR4 and TLR9, respectively (50). But, how Wnt5A signaling is involved in such TLR mediated actin dynamics is unclear. However, this is also not the focus of our study. The results summarized here clearly indicate that optimal levels and patterns of assembled actin are required for killing different bacteria, irrespective of whether these are pathogenic or non-pathogenic. Given the observed effect of Wnt5A signaling on non-pathogens, it is important to understand if commensal bacteria, which have coevolved with the host and are crucial for immune defense, benefit from Wnt5A signaling mediated actin assembly (52–54).

## Data Availability Statement

The original contributions presented in the study are included in the article/**Supplementary Material**. Further inquiries can be directed to the corresponding author.

## Ethics Statement

The animal study was reviewed and approved by CSIR-IICB-Animal Ethics Committee.

## Author Contributions

MS designed research, analyzed data, and wrote the paper. SJ performed research, intellectually contributed to research design, analyzed data, and assisted in writing the paper. SS performed some research and assisted in paper writing. All authors contributed to the article and approved the submitted version.

## Funding

This work was supported by a grant (BT/PR27125/BRB/10/1635/2017) from the Department of Biotechnology, Government of India and institutional funding. SJ was supported by Research Scholar fellowship from CSIR, Government of India and by The Company of Biologist, Journal of Cell Biology. SS was supported by Research Scholar fellowship from CSIR, Government of India.

## Conflict of Interest

The authors declare that the research was conducted in the absence of any commercial or financial relationships that could be construed as a potential conflict of interest.
