# Supplementary material for: Wnt5A-Mediated Actin Organization Regulates Host Response to Bacterial Pathogens and Non-Pathogens
Source: Front Immunol. 2021 Feb 16;11:628191. doi: 10.3389/fimmu.2020.628191 (PMC7921742; doi:10.3389/fimmu.2020.628191)
Supplement: Supplementary file 1 [file DataSheet_1.pdf]

Supplementary Material

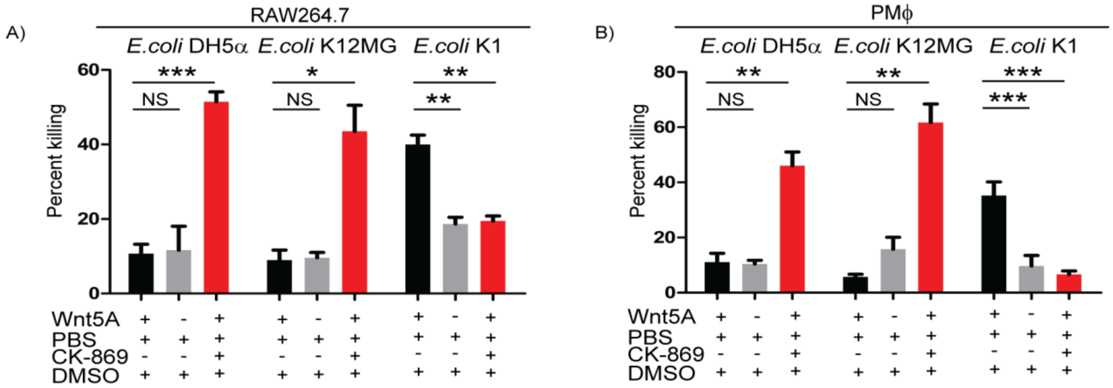

Supplementary Figure 1. ClassII (CK-869) Arp2/3 complex inhibitor alters the fate of pathogenic and non pathogenic bacteria post phagocytosis. Arp 2/3 complex inhibitor CK-869 treatment post infection led to survival of pathogenic *E.coli* K1. On the contrary it induced killing of the non-pathogenic strains *E.coli* K12MG1655 (*E.coli* K12MG) and *E.coli* DH5α in RAW264.7 and peritoneal macrophages (PMϕ) cells upon Wnt5A treatment (6 hrs before infection) as observed in panel A,B. Data represented as mean ± SEM; $*p \leq 0.05$, $**p \leq 0.005$, $***p \leq 0.0005$, NS; Not Significant. AUI: Arbitary Units of Intensity.

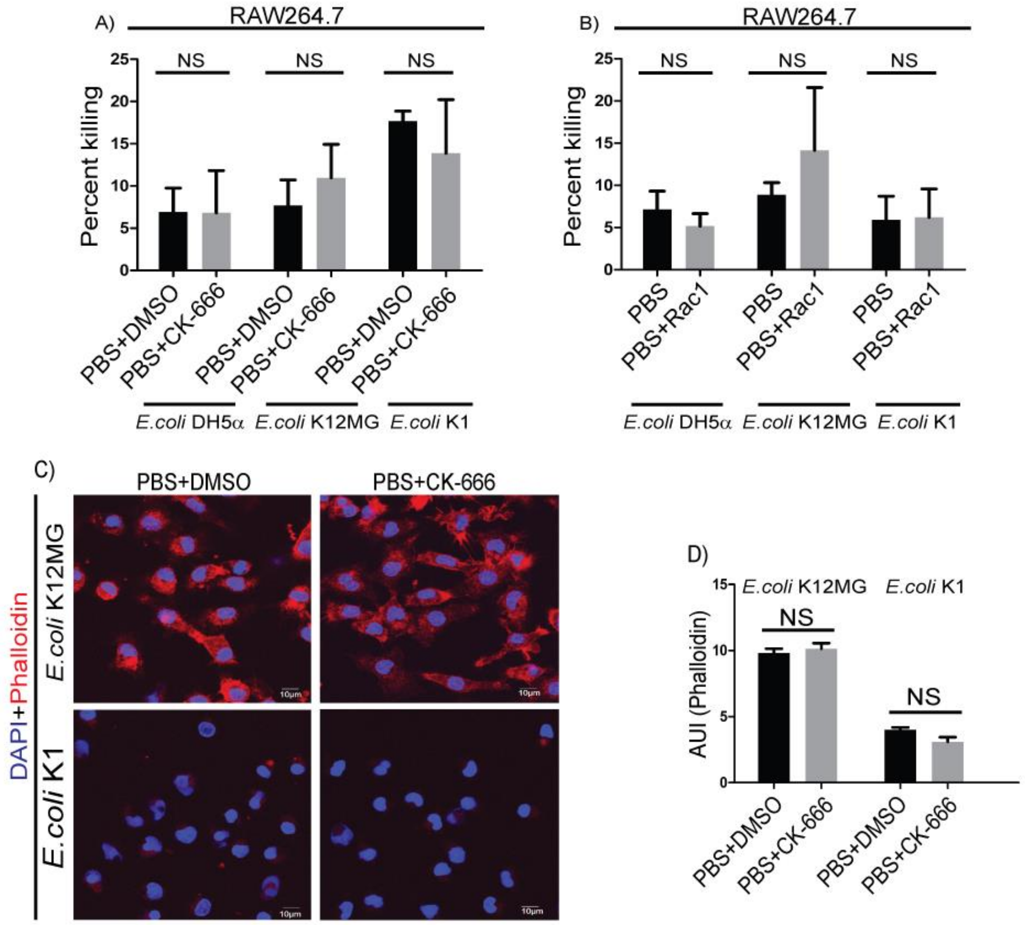

Supplementary Figure 2. Actin assembly inhibitors are not effective in the absence of activation by Wnt5A. Arp 2/3 complex inhibitor class 1 (CK-666) and Rac 1 inhibitor (at 20µM and 15µM concentration respectively) do not have significant effect on the fate of *E.coli* K1 and *E.coli* K12-MG1655 in PBS treated RAW 264.7 as demonstrated by assessment of percent killing after CFU enumeration (A and B), and confocal microsopy of phalloidin stained cells (C and D). Data represented as mean. NS: Not Significant.

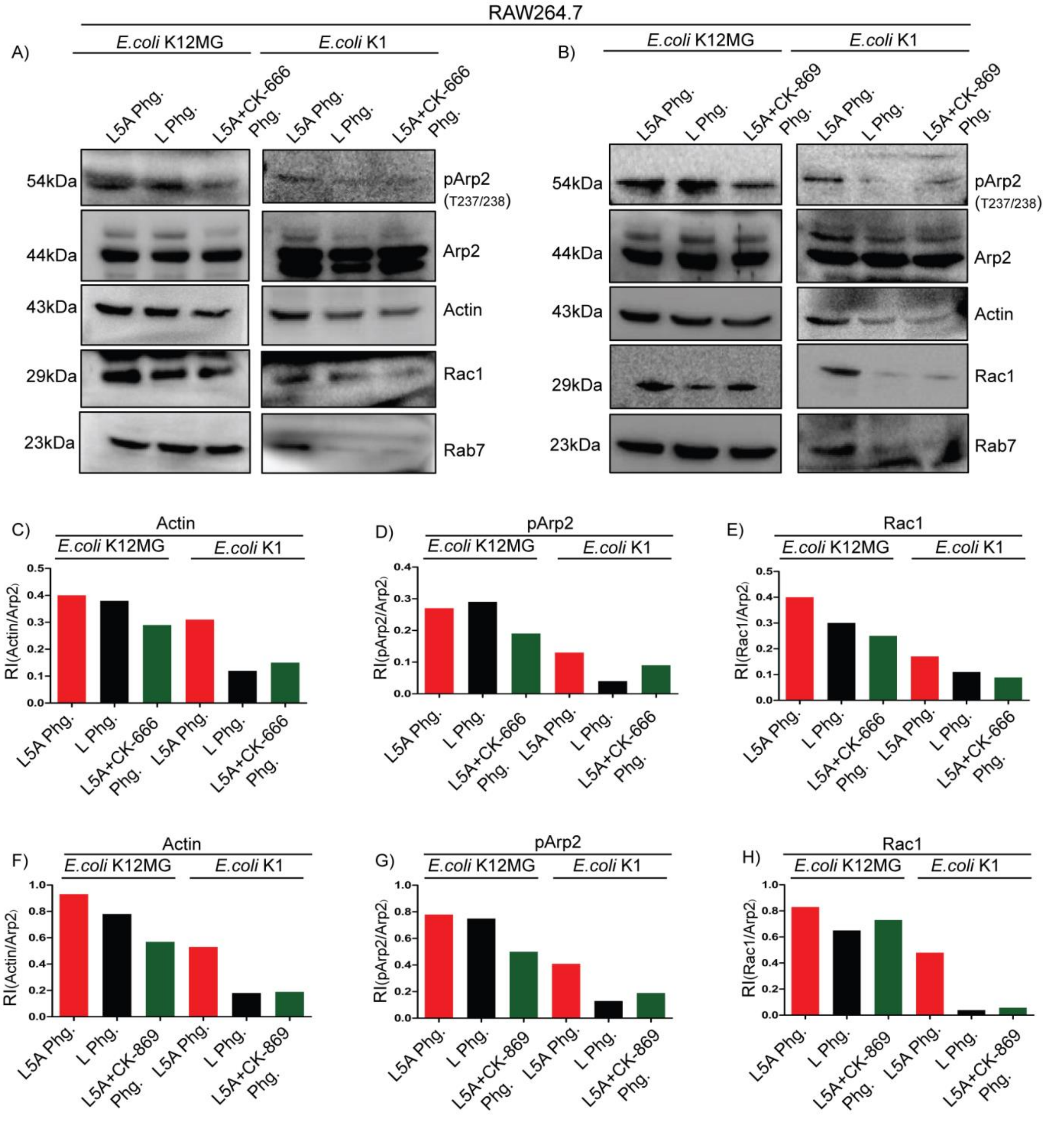

Supplementary Figure 3. <u>Actin assembly inhibitors alter the phagosomal composition of RAW 264.7 macrophages infected with *E. coli* K12-MG and *E. coli* K1 to different extents.</u> The effect of Arp 2/3 complex inhibitors class 1 (CK-666) and class 2 (CK-869) on the Wnt5A induced actin assembly in RAW 264.7 macrophages infected with *E. coli* K12MG and *E. coli* K1was examined at the phagosomal level. After inhibitor treatment the residual actin, pArp2 and Rac1 level with reference to unphosphorylated Arp2 was higher in *E. coli* K12MG infected macrophages than in *E. coli* K1 infected macrophages. Difference in phagosomal composition

was visualized from immunoblots post phagosome isolation (A & B). Densitometric evaluation showing relative intensity (RI) of Actin, p-Arp2 and Rac1 in the phagosome of *E. coli* K12MG1655 infected and *E. coli* K1 infected macrophages with reference to unphosphorylated Arp2 (C-H).

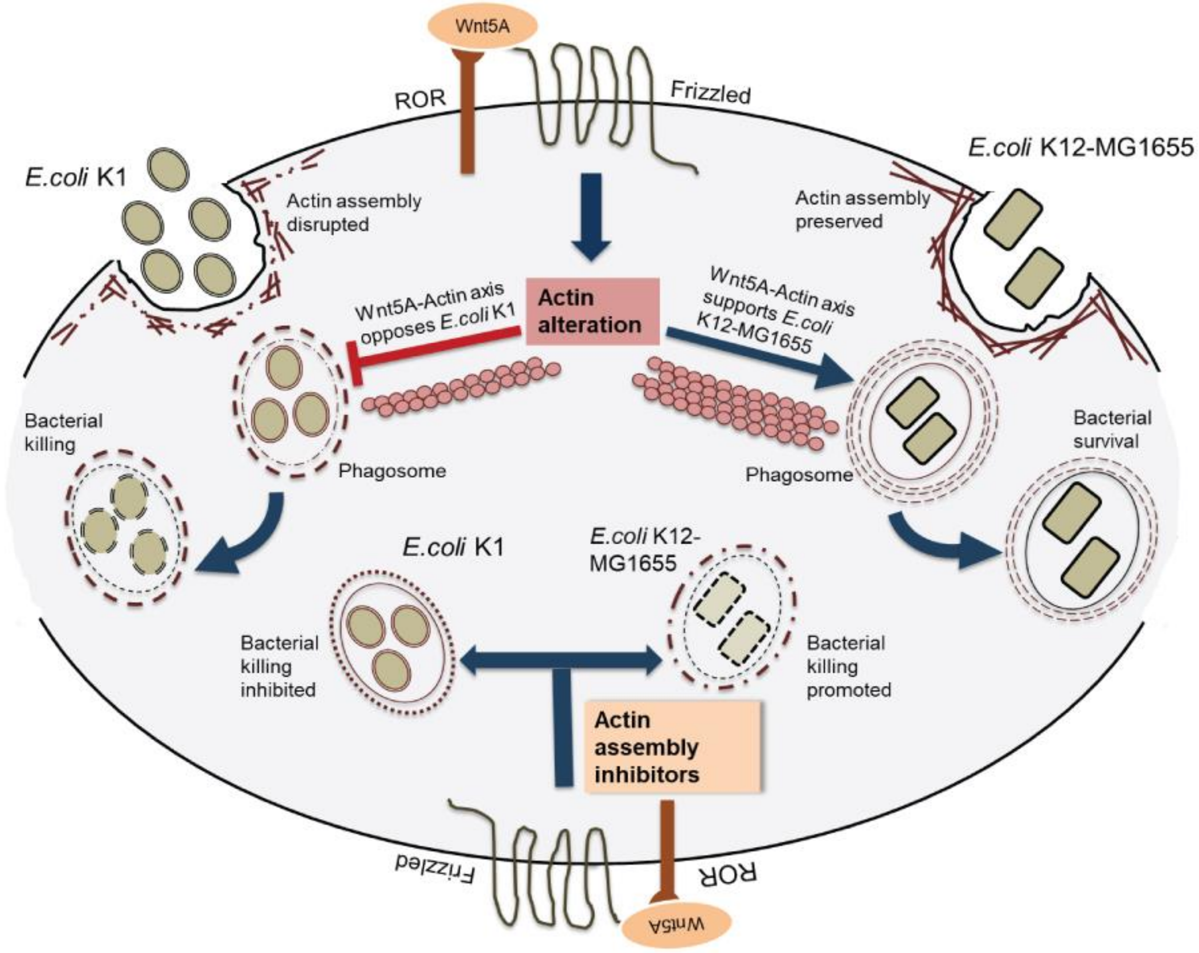

Supplementary Figure 4. Schematic representation of Wnt5A induced actin alterations modulating host response to pathogenic and non-pathogenic bacterial infections. Wnt5A-Frizzled5/ROR signaling induced actin assembly confronts with actin modulations imposed by pathogenic *E.coli* K1 but not non-pathogenic *E.coli* K12-MG1655, shaping their fate inside the macrophages. Actin modulation inhibitors alter the mode of action of Wnt5A signaling towards the pathogenic and non-pathogenic bacterial strains.

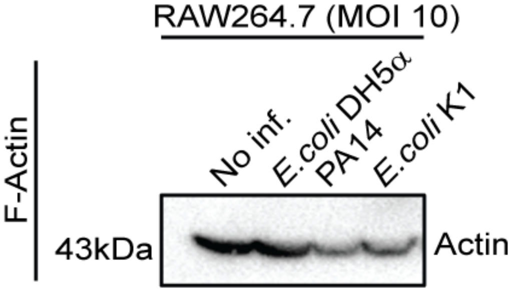

Supplementary Figure 5. *Pseudomonas aeruginosa* (PA14) impairs actin polymerization like pathogenic *E. coli* K1. Imunoblot demonstrating that *Pseudomonas aeruginosa* hindered F actin formation in the cell to avoid killing (Autophagy) induced by Wnt5a signaling (Ref 16). F actin level however was not affected by non-pathogenic *E. coli* DH5α infection.
